# A Medical Bibliography

**Published:** 1891-09-26

**Authors:** 


### PRACTITIONERS' BOOKSHELF.

### A MEDICAL BIBLIOGRAPHY.

Messrs. Lusac and Co., Museum Street, W.C., have recently issued the first quarterly number of an international medical bibliography containing a full list of all works on medicine including important magazine and paper articles published in England and abroad. It is edited by Gustav Ruprech, and the first number is brought down to March, 1891. We shall watch the progress of this publication with great interest, as, if well done, it will meet a distinct want and prove, we believe, widely popular. The subscription price is 6s. nett per annum.

